# Optimized algorithm for multipoint geostatistical facies modeling based on a deep feedforward neural network

**DOI:** 10.1371/journal.pone.0253174

**Published:** 2021-06-22

**Authors:** Jianpeng Yao, Wenling Liu, Qingbin Liu, Yuyang Liu, Xiaodong Chen, Mao Pan

**Affiliations:** 1 The Key Laboratory of Orogenic Belts and Crustal Evolution, School of Earth and Space Sciences, Peking University, Beijing, China; 2 Research Institute of Petroleum Exploration & Development, PetroChina, Beijing, China; 3 Petroleum Exploration and Production Research Institute, Sinopec, Beijing, China; 4 Exploration and Development Research Institute of Changqing Oilfield Branch Company Ltd., PetroChina, Xi’an, China; University of Bradford, UNITED KINGDOM

## Abstract

Reservoir facies modeling is an important way to express the sedimentary characteristics of the target area. Conventional deterministic modeling, target-based stochastic simulation, and two-point geostatistical stochastic modeling methods are difficult to characterize the complex sedimentary microfacies structure. Multi-point geostatistics (MPG) method can learn a priori geological model and can realize multi-point correlation simulation in space, while deep neural network can express nonlinear relationship well. This article comprehensively utilizes the advantages of the two to try to optimize the multi-point geostatistical reservoir facies modeling algorithm based on the Deep Forward Neural Network (DFNN). Through the optimization design of the multi-grid training data organization form and repeated simulation of grid nodes, the simulation results of diverse modeling algorithm parameters, data conditions and deposition types of sedimentary microfacies models were compared. The results show that by optimizing the organization of multi-grid training data and repeated simulation of nodes, it is easier to obtain a random simulation close to the real target, and the simulation of sedimentary microfacies of different scales and different sedimentary types can be performed.

## Introduction

The key link between reservoir characterization and modeling is to explore the mapping relationship between a priori geological model and currently available well log and seismic data. The direct transformation of geological data is limited by the accuracy and quantity of those data. Two-point geostatistical methods based on ‘variogram’ can reflect the correlations between two points in space, but it is difficult for these methods to characterize the spatial structure of complex sedimentary facies. To overcome limitations of conventional approach, a new geostatistical method called multipoint geostatistical (MPG) was developed to simulate geological model based on ‘training image’ [1–4]. Conventional multipoint geostatistical (MPG) methods such as Single Normal Equation SIMulation (SNESIM) and SIMulation with PATterns (SIMPAT) have problems such as unstable training images and discontinuity of the simulated target bodies [5–9]. However, the introduction of training images and search templates into MPG methods has strengthened the learning of a priori geological pattern and the simulation of multipoint associations in space in the process of reservoir facies modeling. Nevertheless, one problem that still urgently needs to be solved is how to accurately capture nonlinear relationships among multiple points in space.

As an important class of artificial intelligence algorithms, neural networks have been proven to possess superior capabilities in expressing nonlinear relationships and have been widely used in image recognition, speech recognition and other fields. Many scholars have also conducted research on the application of neural networks in the field of reservoir facies modeling. Describing the distribution of underground lithofacies or sedimentary facies is an important task in oil and gas exploration. Predecessors have fully studied the interpretation of reservoir lithofacies or sedimentary facies on well logging curves based on deep learning. Some scholars have tried to carry out lithology recognition through neural networks, support vector machines, decision trees, random forests and other machine learning methods, and have achieved certain results [10–14]. As the predecessor of deep learning network, conventional neural network has been widely used in this area [15–20]. With the rise of the third wave of deep learning, deep learning methods have begun to be applied to lithology recognition [21–25].

Many scholars have also done some research on the application of neural networks in the field of 2D or 3D reservoir facies modeling. Some scholars have used neural network to carry out 2D reservoir facies modeling research based on logging petrophysical properties or sedimentary microfacies data [26]. Some scholars have also introduced neural network methods into MPG for 2D sedimentary facies modeling [27]. The introduction of training images in MPG is inspired by the field of artificial intelligence, so the latest developments in the field of deep learning will surely promote the progress of MPG algorithms. Using training images as a priori model, a 3D sedimentary microfacies model was established based on the recursive convolutional neural network method [28]. Some scholars also use the real image and the image generated by the generator to play the game in the discriminator based on the generative confrontation network to achieve the purpose of generating a simulated sedimentary microfacies model. This method is based on a single training image and uses unconditional simulation [29, 30]. Dupont et al. used GAN for the first time to limit the reservoir-scale geological modeling problem to well data. Through the goal-based method, the reservoir-scale 2D model library is generated as training images [31]. These training images display and represent a wide range of sedimentary facies patterns. Zhang et al. extended this technique to establish conditional geological facies models in 3D, and proved that it is superior to advanced multi-point geostatistics in generating more geologically realistic facies models constrained by well data [32]. The above methods have been successfully applied to underground model reconstruction and can be faithful to well information, but they are affected by problems such as accuracy, diversity of generation models, and the amount of loss function calculations [33]. This paper draws on the research ideas of predecessors. Conventional MPG methods can combine multipoint information and use training images as priori geological models. At the same time, neural networks are beneficial for expressing nonlinear relationships [34]. In view of the distinctive characteristics of both classes of methods, this paper presents an optimized MPG reservoir facies modeling algorithm based on a neural network by optimizing the design in terms of the organizational form of the multigrid training data and the repeated simulation of grid nodes. Comparative experiments involving different modeling algorithm parameters, different data conditions and different sedimentary facies structures are reported as examples.

## Mathematical background

### MPG methods

**Fig 1:**
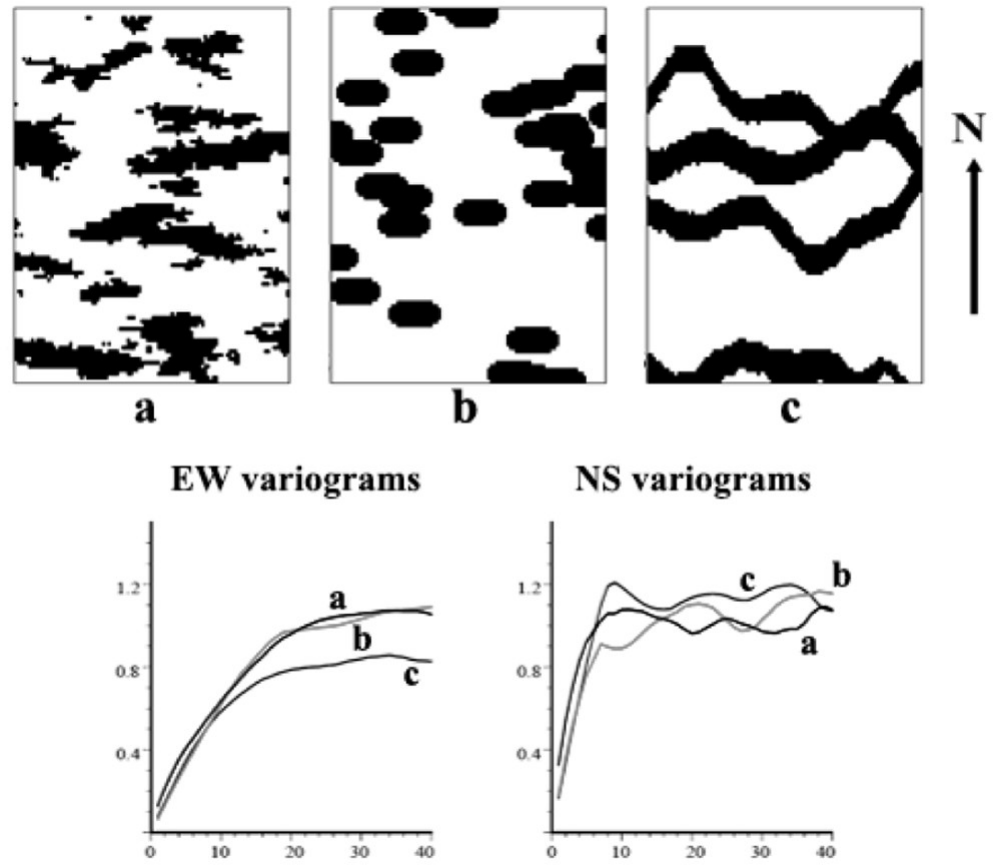
Variation functions of different spatial structures [35].

Variograms are used in conventional two-point geostatistics to measure spatial variability. This method takes advantage of the unbiasedness and optimality of estimation to obtain the optimal solution at the point to be estimated for cross-well prediction. Two-point geostatistics reflect the correlations between two points in space but have difficulty characterizing complex sedimentary facies structures. In Fig 1, panels a-c represent three distinct sedimentary facies structures that nevertheless have very similar east-west and north-south variograms. These examples illustrate that variograms have certain limitations in reflecting spatial anisotropy.

In early work, the most commonly applied MPG methods mainly included simulated annealing with a multipoint statistical objective function, iterative postprocessing based on Gibbs sampling, growth algorithms, Markov Monte Carlo methods based on neural networks and other iterative algorithms. However, due to the harsh convergence conditions and slow calculation speed of these iterative methods, they are greatly restricted in their actual modeling capabilities and difficult to use for practical applications [5].

Strebelle et al. first introduced the concept of a search tree into a probability-based multipoint algorithm (SNESIM). This algorithm uses a dynamic data structure, the "search tree", to store the conditional probability distributions of all training images at once and can then quickly extract the necessary conditions during the process of simulation based on a probability distribution function [2]. Since the introduction of SNESIM, MPG concepts have been widely used. Later, Arpat et al. proposed a similarity-based MPG method (SIMPAT), in which various geological patterns are regarded as the constituent units of an image and reservoir prediction can be treated as an image reconstruction problem [4]. Since the emergence of MPG methods, the above two algorithms have become the most representative approaches.

Many scholars have also optimized and analyzed the multigrid parameters, search template scale, training image generation approaches and other aspects of the above algorithms, thereby improving their accuracy and timeliness to a certain extent [36–42].

### SNESIM—A MPG method based on probability

The core of SNESIM consists of a training image, the data events covered by a search template, and multipoint probability. The SNESIM method considers that the type of facies at any position (s(*u*)) is related to the facies at points around that position (${s(u_{a\prime })=s}_{k_{a\prime }};a\prime =1,\ldots ,n$).

**Fig 2:**
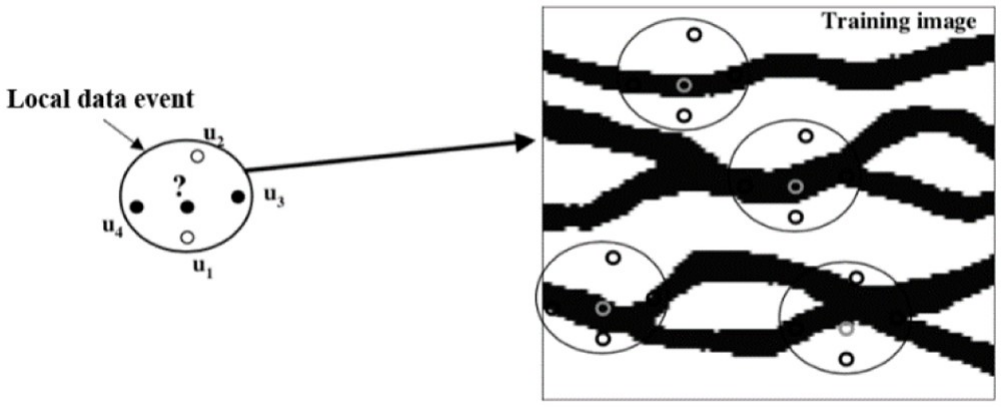
Search template and data events (black represents a channel region, white represents background) [5].

As shown in Fig 2, the search template has a five-point shape. Let a channel point have a value of 1, and let a background point have a value of 0. For the example shown in Fig 2, when the facies values of the surrounding points at the four positions *u*_1_, *u*_2_, *u*_3_ and *u*_4_ are 0, 0, 1, and 1, respectively, *c*(*d*_4_) = 4. According to Eq 1, *c*_1_(*d*_4_) = 3 and *c*_0_(*d*_4_) = 1) Therefore, in this case, the probability of a channel facies value in position u is approximately equal to 3/4. In accordance with this conditional probability approach, sampling is performed to obtain the facies type of each sampled point. Facies modeling can be completed by simulating all unknown points in the target area in turn [5].


$$P\left\{s\left(u\right)=s_{k}|s(u_{a\prime })=s_{k_{a\prime }};a\prime =1,\ldots ,n\right\}=P(u;s_{k}|d_{n})\approx \frac{c_{k}(d_{n})}{c(d_{n})}$$ (1)

Where u represents a spatial position; s(*u*) represents the facies value of u; $s(u_{a\prime })=s_{k_{a\prime }};a\prime =1,\ldots ,n$ represent the facies values of n points around position u; k represents the type of sedimentary facies; *d*_*n*_ represents a data event covered by the search template at position u; *c*_*k*_(*d*_*n*_) represents the number of repeated data events with facies type k at u.

A "search tree", a dynamic data structure, can store the conditional probability distributions of all training images at once, allowing the conditional probability distribution function to be quickly extracted during the simulation process. Since the initial proposal of SNESIM, its computational efficiency has been greatly improved.

### Deep feedforward neural network (DFNN)

DFNN is one of the most basic and important network structures for artificial intelligence neural networks. Its goal is to predict output data that are as close as possible to the real output data (in the output layer) when input data enter the network (in the input layer). The hidden layers in the network structure determine the accuracy of the output data. Almost all deep learning algorithms can be fairly simply described as a combination of a specific data set, cost function, optimization process and model. The training and optimization process for a neural network is as follows:

(1) Prepare the data set and network structure:
  Perform data set preparation (training set and validation set) and network structure preparation (number of network layers, number of neurons in each layer, activation function, cost function, learning rate, regularization coefficient, number of epochs, etc.).
(2) Train:
  ① Initialize the weights w and biases b of each layer and each neuron.
  ② Draw samples from the training set as input, and output the results through each layer of the neural network.
  ③ Analyze the errors between the output and the true values.
  ④ Update all w and b using a feedback mechanism.
  ⑤ Repeat steps 1–4 until all samples from the training set have been used as input.
  ⑥ Calculate the total error value and analyze the accuracy of the network model on the verification set.
  ⑦ Repeat steps ②-⑥ until the initially set number of epochs is reached.
(3) Adjust and optimize:
  Adjust the various hyperparameters of the network based on the total error value and correct output rate in each epoch. Whether overfitting can be used to judge the training status of the neural network model. Overfitting means that the model has poor prediction performance for unknown samples. At present, in the case of limited data conditions, there are many methods to prevent overfitting, such as early stopping or regularization.
  Since we cannot directly apply the trained neural network to another new task, at the same time, the current general difficulty is the need to design a specific neural network for a specific task. Therefore, the application of such deep learning methods in reservoir modeling has certain limitations. Transfer learning developed in recent years is an important research direction of machine learning. Transfer learning can solve the problem of small samples and improve modeling efficiency. The goal of transfer learning is to apply knowledge or patterns learned in a task to different but related tasks.

## Optimized MPG algorithm based on a DFNN

### Background

In the MPG approach, an a priori geological model is expressed in the form of training images. Such a model is a quantitative representation of the target body’s trend of morphological change, the positional relationship between the target body and the background, and the sedimentary characteristics and background of the target area. According to Tobler’s First Law of Geography, "Everything is related to everything else, but near things are more related to each other"; thus, it can be considered that the attribute characteristics of a certain location are closely related to the attribute characteristics of its neighboring points [43]. A search template can therefore be introduced in MPG analysis to capture the attribute characteristics and location characteristics of data events covered by that template. The attribute features of a certain location can be considered to be related to the attribute and location features of the corresponding data event centered on that point. However, due to the strong heterogeneity of the continental sedimentary system, it is not easy to draw a clear statistical relationship between the features of a point and those of its surroundings. The feature extraction and nonlinear relationship expression capabilities of neural networks provide a new way to address such problems. The neural network approach allows a computer to acquire knowledge from experience and understand the world in accordance with a "deep" conceptual system. Each concept can be defined in terms of several relatively simple concepts. This is the main feature of deep learning in artificial intelligence [9]. The computer can gain "experience" from training images that represent a priori geological patterns and further acquire "knowledge" related to the attribute characteristics of a certain location so as to simulate output that is as close as possible to a given a priori geological model or even a real geological model.

### Optimized algorithm

The optimized algorithm presented in this paper draws on previous researchers’ major ideas regarding the combination of multipoint geostatistics and neural networks [11]. In the data preparation and model simulation stages, the organization of the multigrid training data set and the repeated simulation of grid nodes are optimized.

**Fig 3:**
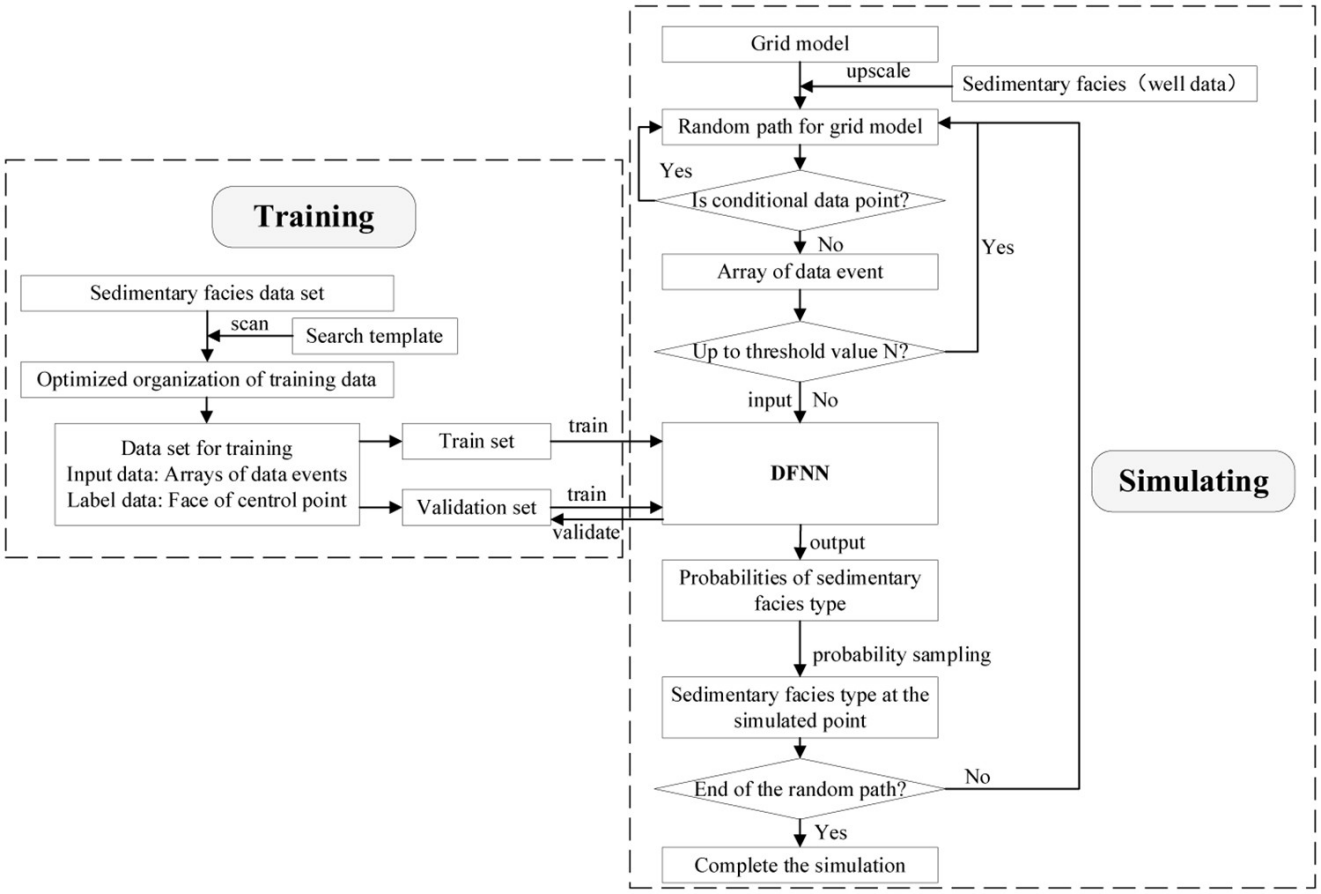
Overall procedure design.

**Fig 4:**
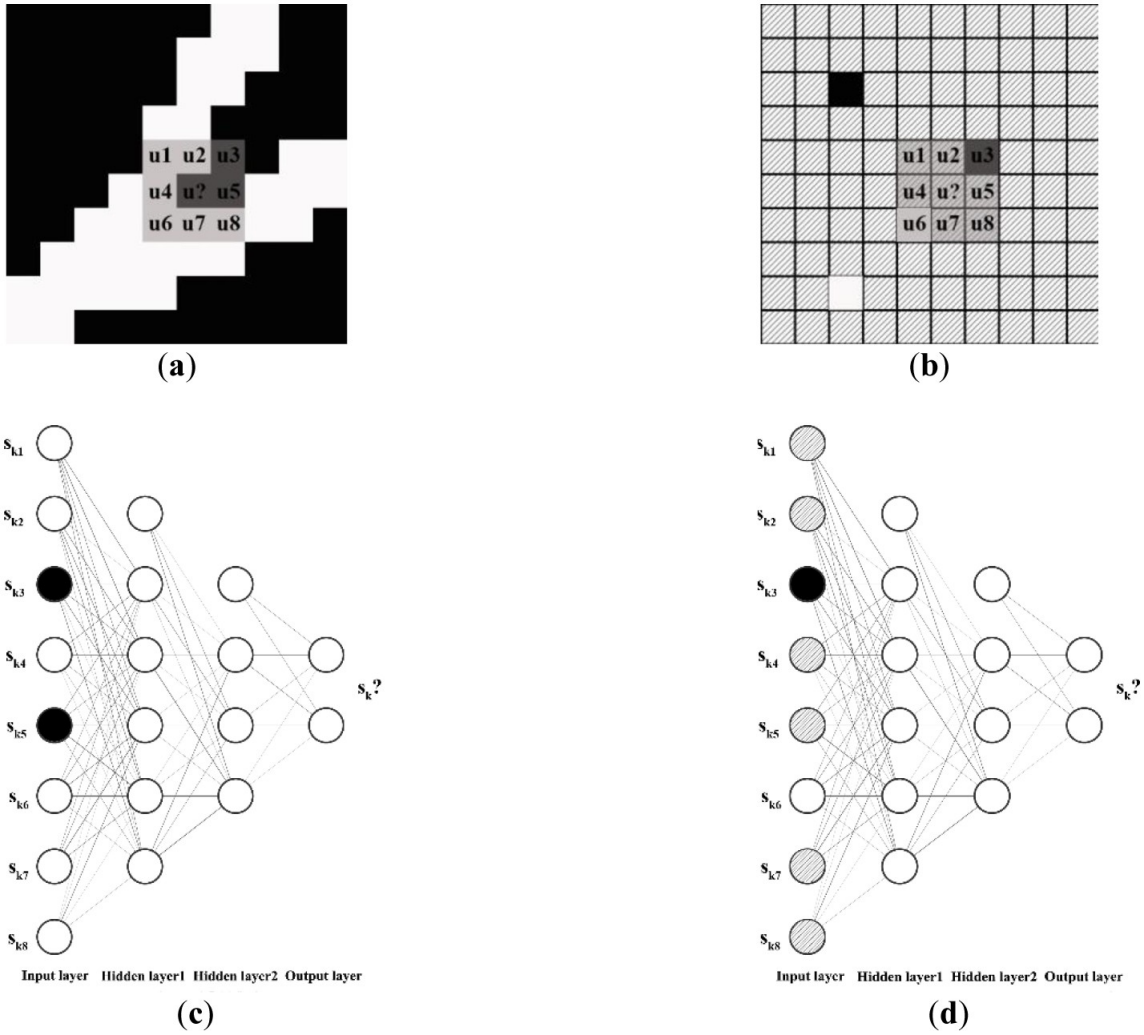
Schematic illustration of facies modeling: (a) training; (b) simulation; (c) training data; (d) simulated data (a white node represents a channel facies value, a black node represents a background value, and a shaded node represents a null value).

**Fig 5:**
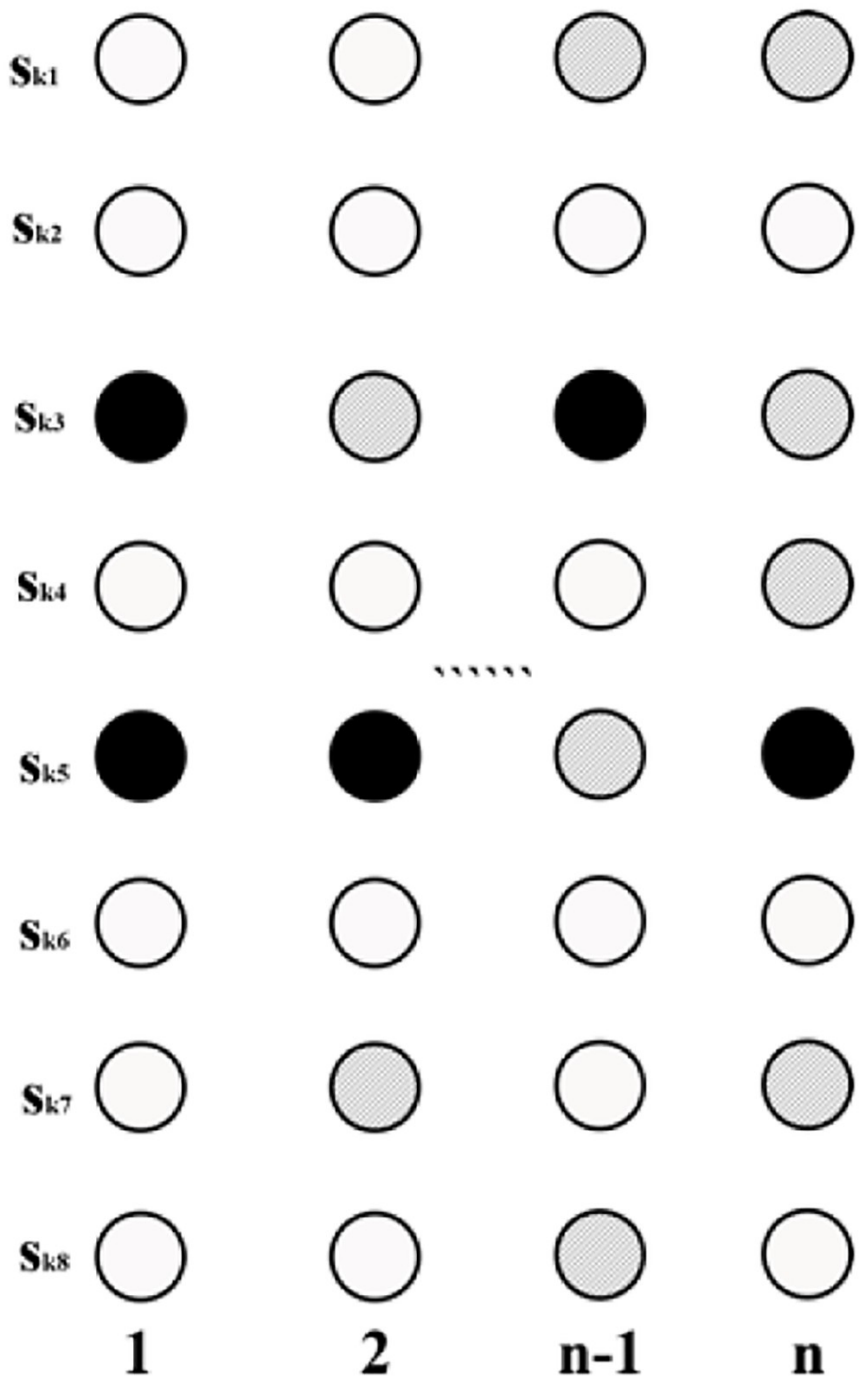
Optimized training data organization (a white node represents a channel facies value, a black node represents a background value, and a shaded node represents a null value).

**Fig 6:**
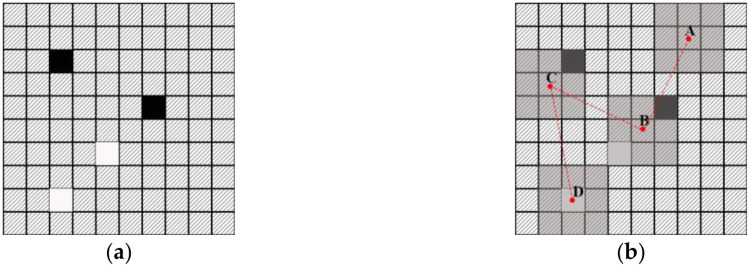
Repeated simulation of grid nodes (the red dotted line represents a random path).

The workflow of optimized algorithm is shown in Fig 3 and the specific steps are as follows:

(1) Data set preparation:
  ① Generation of training images:
    Training images can be hand-drawn or generated with the help of petroleum geostatistical software based on the actual geological characteristics of the study area.
  ② Definition of the specifications of the search template and the form of the multigrid:
    The sublevels of the multigrid are defined as required, and the search templates for each level of the multigrid are homogeneous.
  ③ Optimization of the organization of the multigrid training data:
    It is necessary to establish a separate training data set for each level of the multigrid. As shown in Fig 4a, a 3*3 search template is taken as an example to illustrate the process of optimizing the organization of the training data. In the conventional method, the data event array *S*_*kn*_ at position u must consist entirely of channel and/or background values, but during the actual simulation process (Fig 4b), *S*_*kn*_ may contain multiple null points. Thus, in this paper, the organization of the data set at different multigrid levels is optimized in accordance with the characteristics of data events during the training and simulation processes. Simulation is performed from the g-th (coarsest) level of the multigrid to the first (finest) level of the multigrid. The scale of the search template is gradually reduced accordingly; thus, the number of null points in *S*_*kn*_ is also gradually reduced during the simulation process.
    Therefore, in view of the different numbers of null points in *S*_*kn*_ in different levels of the multigrid, optimal null point insertion is performed on the training data set at different multigrid levels. Taking the first level of the multigrid as an example, after the simulation of the other multigrid levels, there will be no more than 4 null points in *S*_*kn*_. Therefore, for optimal organization of the training data set, fewer than 4 null value points are randomly inserted into each data event array to meet the particular requirements of the training data for this level of the multigrid.
  ④ Neural network training:
    The established training data set (see Fig 5) is loaded into the neural network for training, and the trained neural network parameters are obtained for subsequent simulation.
(2) Simulation:
  The model to be simulated is formatted, and well point data are used as condition data to perform the simulation.
  ① Simulation based on the multigrid:
    A path traversing the current level of the multigrid is randomly generated. The simulation process starts at the first grid node on this path, and the corresponding data event is obtained. As shown in Fig 3b and 3c, where a 3*3 search template is taken as an example, the sedimentary facies type array *S*_*kn*_(*n* = 1, 2, …, 8) corresponding to *u*_*n*_(*n* = 1, 2, …, 8) is used as the input to the neural network. Then, the sedimentary facies type *S*_*k*_ of the center point u that is simulated by the neural network is output.
  ② Probability sampling:
    The sedimentary facies type at the simulated point is directly sampled based on the probabilities output by the neural network to obtain the final simulated value at this point.
  ③ Repeated simulation of the grid nodes for optimization of the results:
    In the initial stage of the simulation, the input data *S*_*kn*_ may contain only a few known points, while the rest are all null points. In the conventional method, each point is simulated only once, which can easily lead to simulation failure because of too many null points. In this paper, an optimization method is designed in which the simulation process is repeated at certain points. A user-defined threshold value N is set for triggering repeated node simulation. When the number of null points in *S*_*kn*_ is fewer than N, probability sampling can be performed. Otherwise, this point is added back to the random path for repeated simulation.
    Taking a 3*3 search template as an example, Fig 6b shows a random path from point A to point D. A threshold of N = 8 is set, meaning that the data event currently covered by the search template can be simulated if it contains at least one known point. The simulation process starts at point A; since all of the values around this point are empty, point A is added back to the random path. By contrast, the numbers of null points in the data events at points B and C are below the threshold value, thus meeting the conditions for simulation. After simulation, these two points can be added to the condition data set. Point D itself is a conditional data point, so there is no need to simulate this point. In this way, all points along the random path are simulated.
  ④ Traversal of all grid nodes to be simulated on the random path to complete a random simulation of the model.

## Case studies

Spatial patterns consistent with the meandering river model and the delta plain model, which have relatively complex structures, are commonly encountered in oil and gas reservoirs. Therefore, to verify the validity and practicability of the proposed DFNN-based MPG facies modeling algorithm, these two models are considered as examples for analysis. Simultaneously, comparative experiments of model simulations based on different data conditions and modeling algorithm parameter conditions are carried out. Based on these comparisons, the optimal parameter conditions of the modeling algorithm are summarized. The reconstruction of the training images is the main basis for evaluating the effectiveness of geostatistical algorithms; thus, a corresponding analysis and comparison are carried out.

**Fig 7:**
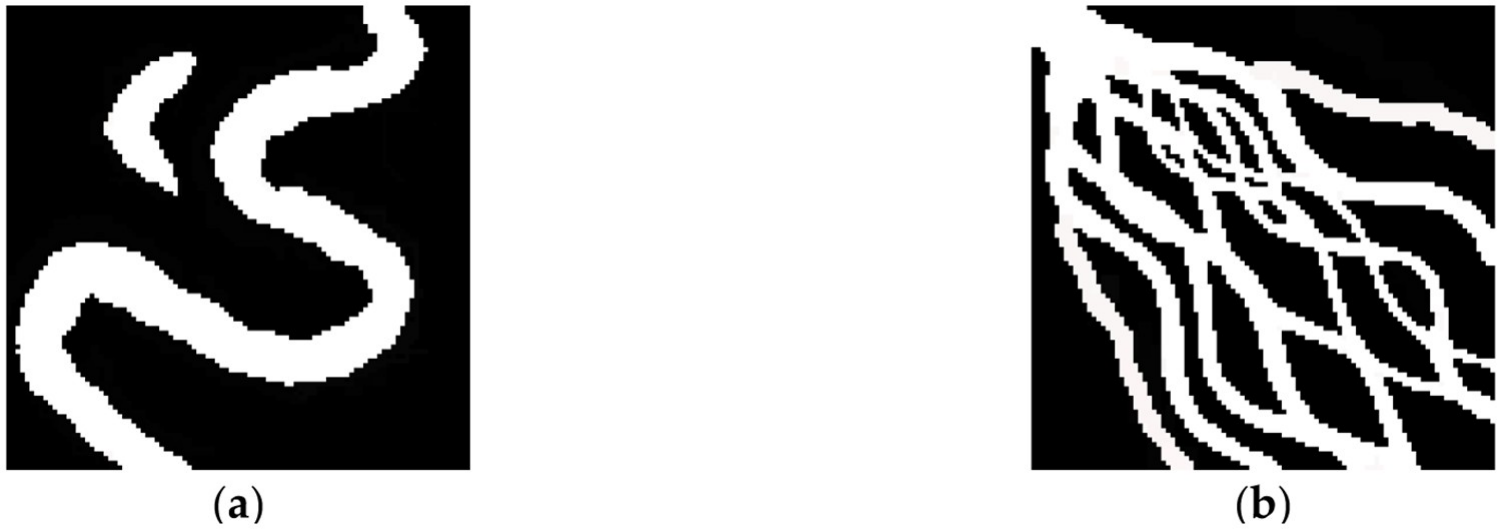
Training image data sets: (a) meandering river model (100*100); (b) delta plain model (100*100).

The training data sets for the sedimentary models considered in this research are shown in Fig 7. Fig 7a shows the training image for the meandering river model (including a main channel and an oxbow lake), and Fig 7b shows the training image for the delta plain model (including distributary channel facies and interdistributed channels). Both models have dimensions of 100*100. First, a variety of simulation methods (the conventional DFNN-based MPG method, the SNESIM method and the optimized DFNN-based MPG simulation method) are considered in comparative experiments conducted on training image 6a. Then, simulation experiments carried out using the optimized algorithm for the delta plain model (Fig 7b), which has a more complex structure, are presented. The parameter settings of the network are shown in Table 1. The code and program used in this article are completed based on Python 3.6.11 and TensorFlow 1.10.0.

**Table 1:** Neural network parameter settings.

Number of layers	7
Number of input nodes	Determined by the number of nodes in the data event
Number of output nodes	Determined by the number of label data types
Number of nodes in hidden layers	[50, 30, 20, 15, 10]
Activation function	leaky-relu(α: 0.01)
Optimization algorithm	Adam (learning_rate: 0.001; *β*_1_: 0.9; *β*_2_: 0.999; ε: 1*e*^−8^)
Initialization strategy	glorot_uniform
Data split	Training data (70%), Validation data (20%), Test data (10%)

The setting of neural network is complicated, mainly including the selection and setting of hyperparameters such as the number of layers of the neural network, the number of neurons, the activation function, the cost function, the optimization algorithm, and the initialization method. Neural network settings for specific tasks may need to be obtained through constant experimentation. For example, too many hidden layers may lead to overfitting, on the contrary, too few hidden layers may not be able to perform nonlinear fitting. For another example, some activation functions may cause the disappearance of gradients or the death of neurons. Leaky-relu is currently the more commonly used activation function. At the same time, the influence of other optimization algorithms such as gradient descent must be integrated. For example, compared to SGD, BGD and MBGD, the Adam method combines the advantages of Momentum and RMSprop, and is a commonly used algorithm for adaptive learning rate.

### A case study of the meandering river model

**Fig 8:**
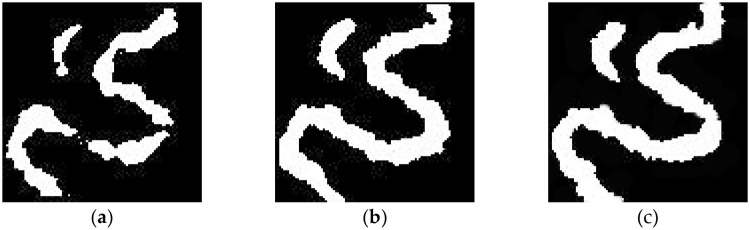
Comparison of the results obtained with different quantities of conditioning data: (a) 100; (b) 200; (c) 300.

First, the optimized algorithm was used to simulate the meandering river model under the control of different numbers of conditional data points. As shown in Fig 8a, with only 100 conditional data points, the basic trends of the main channel and oxbow lake are already clearly visible. However, because of the limited amount of conditional data, the riverbed width varies greatly, and there are many breakpoints. When the number of conditional data points is doubled (Fig 8b), the main channel and oxbow lake are continuous and complete. Overall, the shape and structural characteristics of the simulation results are basically consistent with those of the target model. When the number of conditional data points is further increased (Fig 8c), no obvious improvement in the simulation effect is achieved. Therefore, for the meandering river model at this scale, the goal of reconstructing the target model can be basically achieved with approximately 200 conditional data points. Thus, 200 conditional data points were used in the following comparative experiments.

In the section ‘Optimized algorithm’, the optimization of the conventional method in terms of the organization of the training data and the repeated simulation of grid nodes has been explained in detail. Accordingly, multipoint statistical simulations based on a DFNN were performed for the training image presented in Fig 7a as an example.

**Fig 9:**
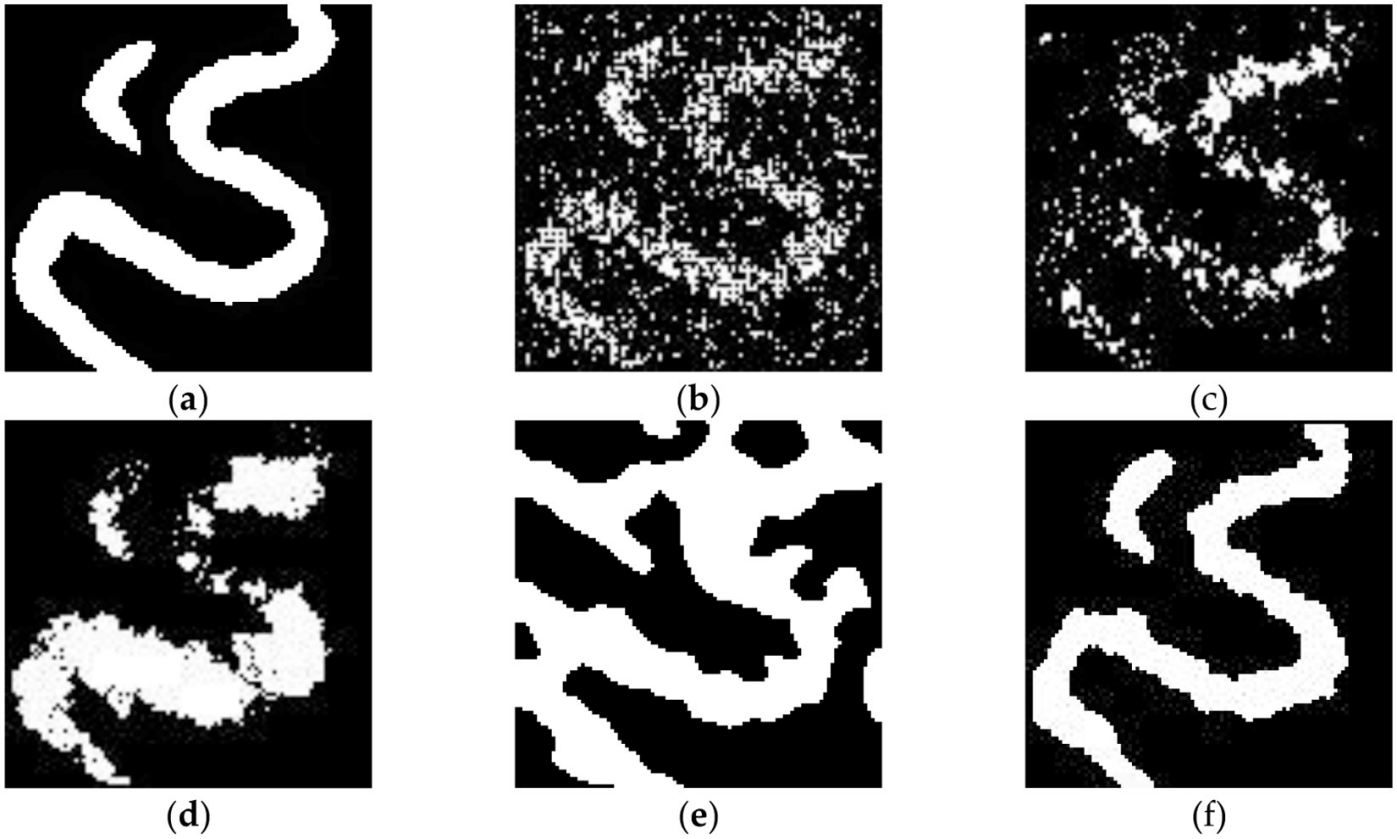
Random simulations of the channel model: (a) target model; (b) realization using the conventional MPG method based on a DFNN; (c) realization using the repeated simulation method; (d) realization with the optimized training data organization; (e) realization using SNESIM; (f) realization using the optimized MPG method based on a DFNN.

Fig 9b shows the simulation results of the conventional MPG method based on a DFNN. Without either the optimized training data organization or the repeated simulation of nodes, not only do the results contain many scattered points, but the morphological structure of the main channel is only vaguely visible. Fig 9c shows the results obtained when only repeated node simulation is performed without the optimized training data organization. The number of scattered points is significantly reduced, and the number of breakpoints in the main channel is slightly reduced. Fig 9d shows the results obtained with the optimized organization of the training data but without the repeated simulation of grid nodes. In this case, the scattered points in the simulation results are basically eliminated, but there are still breakpoints in the main channel as well as some anomalous widening. Fig 9e shows the results of SNESIM. Although there are no scattered points or breakpoints in these simulation results, the shape and structure of the oxbow lake and main channel are obviously abnormal. These simulation results are somewhat distorted. Fig 9f shows the simulation results of the optimized algorithm with both the optimized form of the training data and repeated node simulation. In this case, the morphology and structure of the main channel and oxbow lake are clearly reproduced.

The above experiments show that MPG facies modeling based on a DFNN with optimized training data organization and repeated node simulation has obvious advantages over both the conventional DFNN-based MPG simulation method and the SNESIM method.

### A case study of the delta plain model

To further test the optimized DFNN-based MGP algorithm, the more complex delta plain model was used for simulation experiments. In constructive deltas, the distribution of the distributary channel sand bodies is very complicated due to frequent divergence and integration of the distributary channels and large changes in width (Fig 7b).

**Fig 10:**
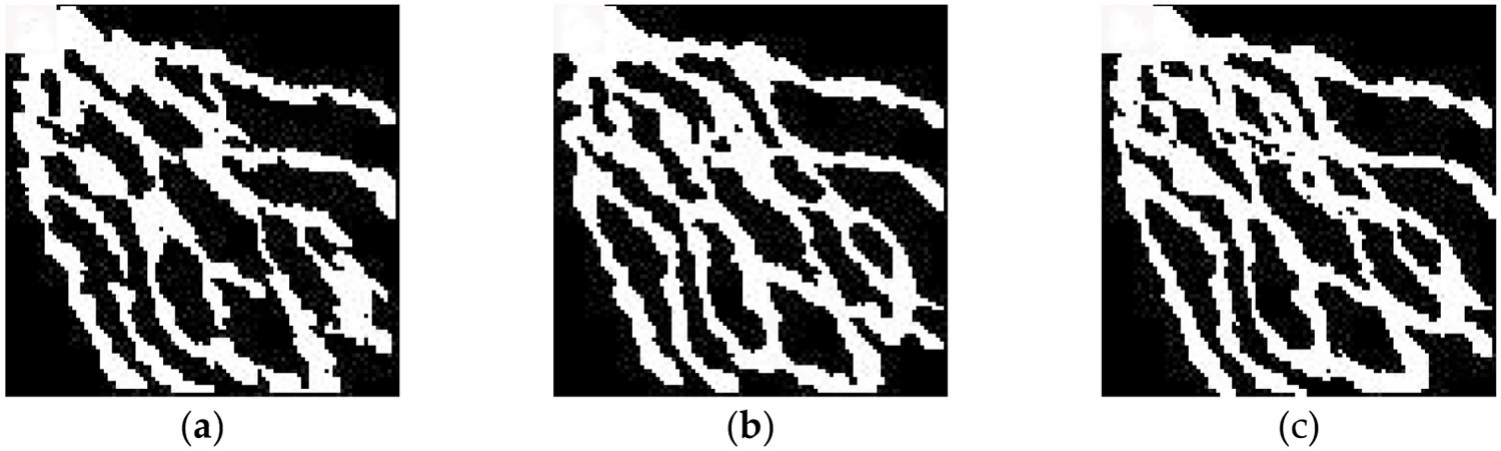
Comparison of the results obtained with different numbers of conditional data points: (a) 350; (b) 450; (c) 550.

First, the optimized algorithm was used to simulate the delta plain model under the control of different numbers of conditional data points. As shown in Fig 10, with 350 conditional data points (Fig 10a), the basic structure of the main branch channels of the distributary channel facies is already clear. However, because of the limited number of conditional data, there are several breakpoints in the simulation results, and some secondary channels are not simulated. When the number of conditional data is increased to 450 (Fig 10b), the channel shape is obvious, the primary and secondary distributary channel facies are essentially all simulated, and the channel width tends to be more uniform and smoother. The results are almost consistent with the target model, basically achieving the purpose of reconstruction. When more conditional data points are added (Fig 10c), the reconstruction effect is not significantly improved. Therefore, for the delta plain model at this scale, the goal of reconstructing the target model can be basically achieved with approximately 450 conditional data points.

**Fig 11:**
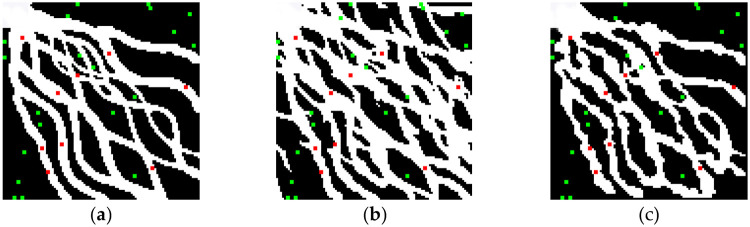
Stochastic realizations of the complex sedimentary model: (a) target model; (b) realization using SNESIM; (c) realization using optimized MPG facies modeling based on a DFNN.

Based on the above results, 450 conditional data points were used in the following comparative experiments. Fig 11b and 11c show the simulation results obtained for the delta plain model using SNESIM and the optimized method presented in this paper under the same data conditions. As shown in Fig 11b, the simulation results of SNESIM capture the overall trend of the northwest-southeast river channel, but an oversimulation phenomenon is observed in which many non-existent secondary branch channels are simulated between the main branch channels. By contrast, as shown in Fig 11c, the simulation results obtained using the optimized method contain neither scattered points nor breakpoints and show no oversimulation, basically reproducing the target model.

To quantitatively verify the accuracy of the simulation results, 30 verification wells (not used in the simulation process) were randomly selected for testing in the delta plain model simulation experiments. The experimental results show that the accuracy of the simulation results based on the SNESIM method and optimized method is 77% and 97%, respectively.

In summary, this example proves that MPG facies modeling based on a DFNN with optimization in two aspects, i.e., training data organization and repeated node simulation, can successfully simulate complex reservoir facies models with a simulation effect that is significantly better than that of conventional methods.

## Conclusions

An optimized organizational form for multigrid training data and a mechanism for the repeated simulation of grid nodes have been designed in this paper, thus establishing an optimized algorithm for MPG facies modeling based on a DFNN. Comparative experiments have been carried out using the meandering river model and the delta plain model.

1. The experimental results show that the optimized multigrid training data organization method can avoid the problem of scattered points encountered in conventional simulation methods but has no obvious effect on the breakpoint problem. However, with the further optimization of repeated node simulation, the breakpoint problem of conventional methods can also be mitigated to a certain extent.
2. With the proposed dually optimized design (with optimized training data organization and repeated node simulation), it is easier to obtain random simulation results that are close to the real target model. The proposed method can also successfully simulate sedimentary facies models of different scales and with different complex structures.
3. To further improve the modeling accuracy, seismic data could also be applied as constraints. This would allow the joint constraints represented by well data and seismic data to be associated with sedimentary facies in order to simulate a three-dimensional reservoir sedimentary facies model that is closer to the actual geological model.

## Supporting information

S1 Fig High resolution figures. All Figures of the whole paper along with extra figures are listed.
(PDF)
