# Supplementary material for: Optimized algorithm for multipoint geostatistical facies modeling based on a deep feedforward neural network
Source: PLoS One. 2021 Jun 22;16(6):e0253174. doi: 10.1371/journal.pone.0253174 (PMC8219166; doi:10.1371/journal.pone.0253174)
Supplement: S1 Fig — All Figures of the whole paper along with extra figures are listed. (PDF) [file pone.0253174.s001.pdf]

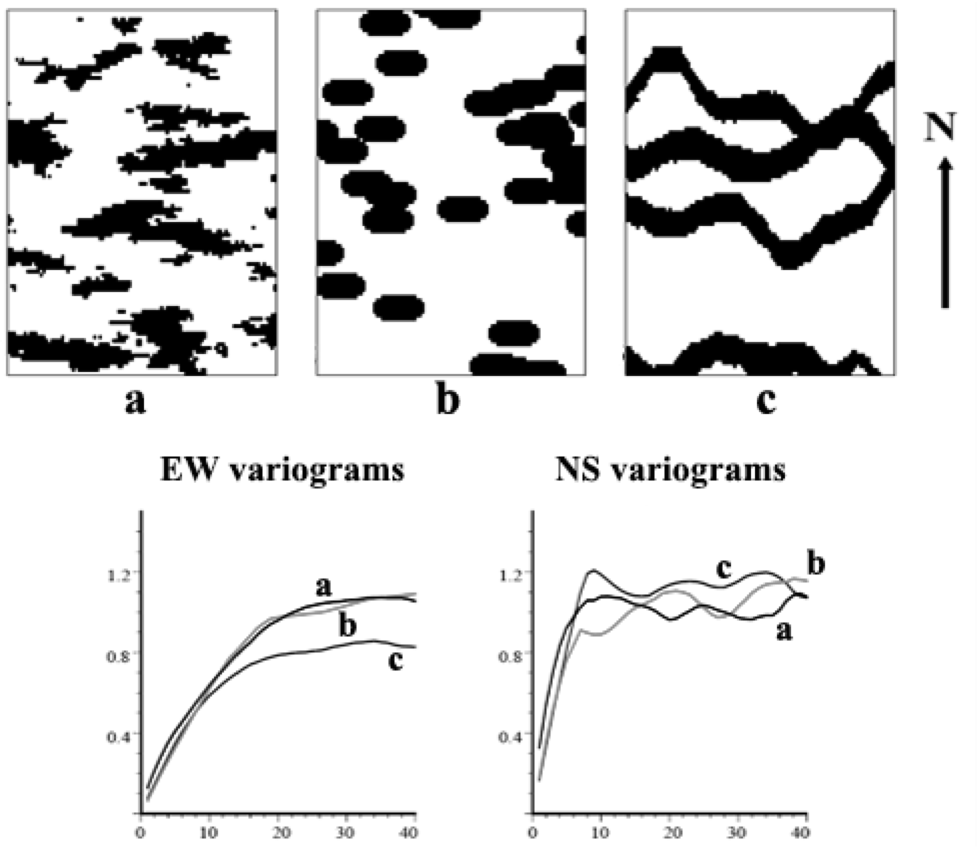

Fig 1 Variation functions of different spatial structures

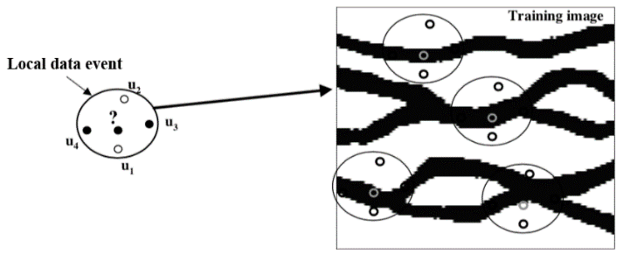

Fig 2 Search template and data events (black represents a channel region, white represents background)

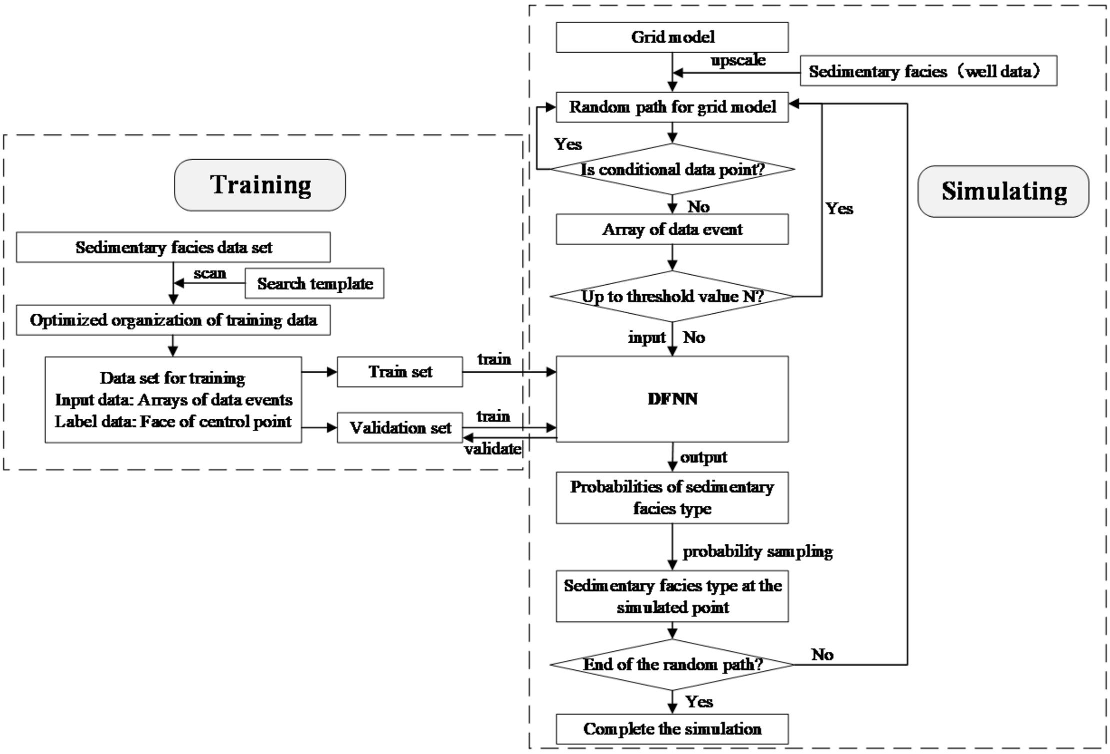

Fig 3 Overall procedure design

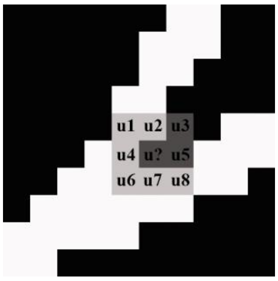

Fig 4a Schematic illustration of facies modeling: (**a**) training (a white node represents a channel facies value, a black node represents a background value, and a shaded node represents a null value)

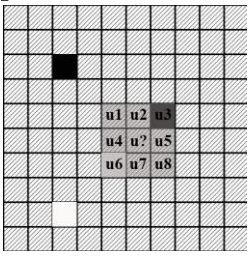

Fig 4b Schematic illustration of facies modeling: (**b**) simulation (a white node represents a channel facies value, a black node represents a background value, and a shaded node represents a null value)

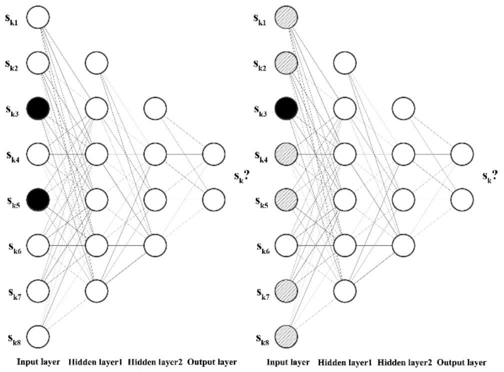

Fig 4c Schematic illustration of facies modeling: (**c**) training data (a white node represents a channel facies value, a black node represents a background value, and a shaded node represents a null value)

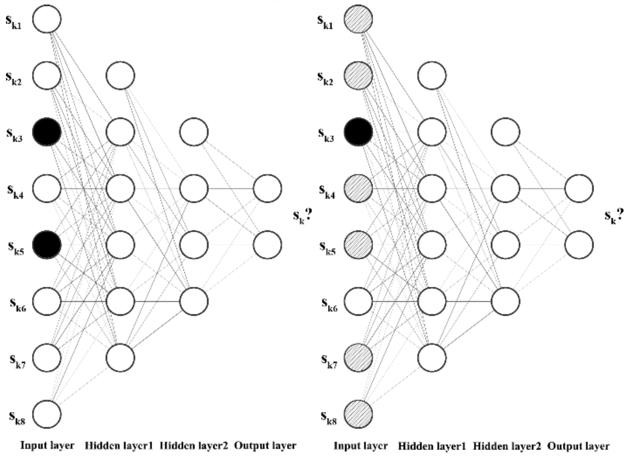

Fig 4d Schematic illustration of facies modeling: (**d**) simulated data (a white node represents a channel facies value, a black node represents a background value, and a shaded node represents a null value)

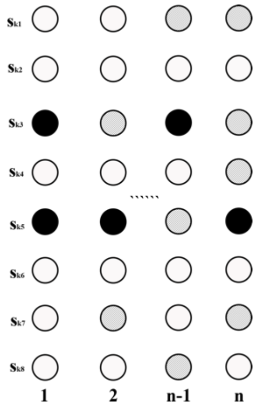

Fig 5 Optimized training data organization (a white node represents a channel facies value, a black node represents a background value, and a shaded node represents a null value)

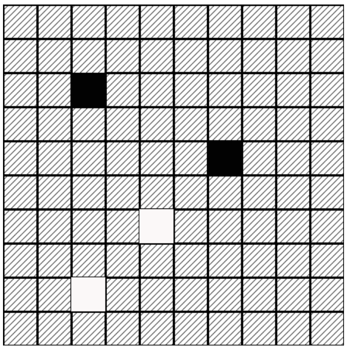

Fig 6a Repeated simulation of grid nodes (the red dotted line represents a random path).

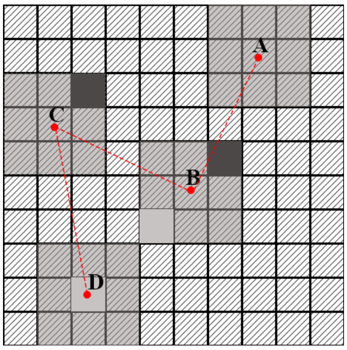

Fig 6b Repeated simulation of grid nodes (the red dotted line represents a random path).

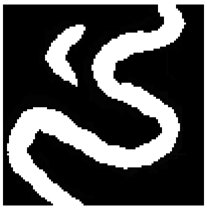

Fig 7a Training image data sets: (a) meandering river model (100*100)

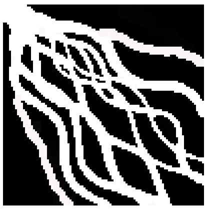

Fig 7b Training image data sets: (b) delta plain model (100*100)

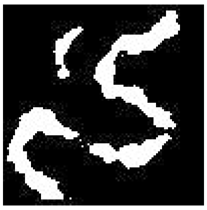

Fig 8a Comparison of the results obtained with different quantities of conditioning data: (a) 100

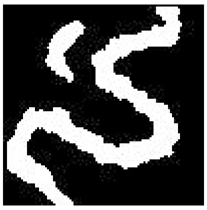

Fig 8b Comparison of the results obtained with different quantities of conditioning data: (b) 200

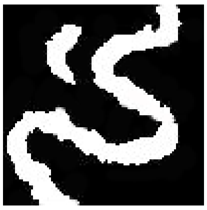

Fig 8c Comparison of the results obtained with different quantities of conditioning data: (c) 300

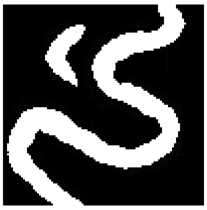

Fig 9a Random simulations of the channel model: (a) target model

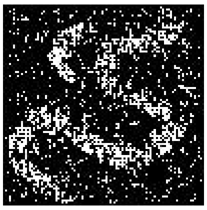

Fig 9b Random simulations of the channel model: (b) realization using the conventional MPG method based on a DFNN

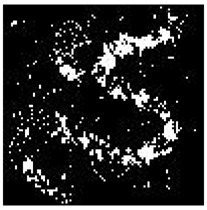

Fig 9c Random simulations of the channel model: (c) realization using the repeated simulation method

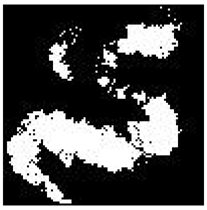

Fig 9d Random simulations of the channel model: (d) realization with the optimized training data organization

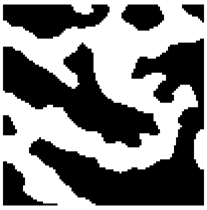

Fig 9e Random simulations of the channel model: (e) realization using SNESIM

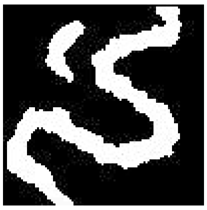

Fig 9f Random simulations of the channel model: (f) realization using the optimized MPG method based on a DFNN

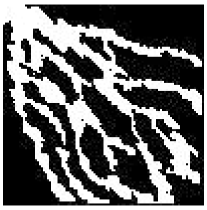

Fig 10a Comparison of the results obtained with different numbers of conditional data points: (a) 350

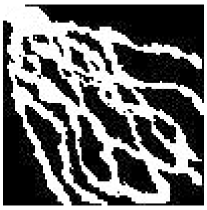

Fig 10b Comparison of the results obtained with different numbers of conditional data points: (b) 450

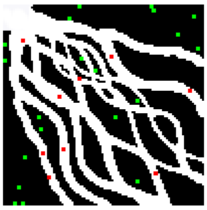

Fig 10c Comparison of the results obtained with different numbers of conditional data points: (c) 550.

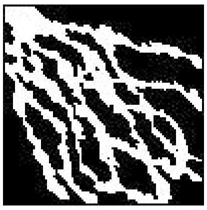

Fig 11a Stochastic realizations of the complex sedimentary model: (a) target model

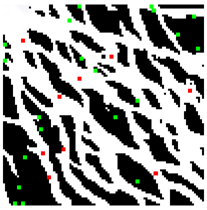

Fig 11b Stochastic realizations of the complex sedimentary model: (b) realization using SNESIM

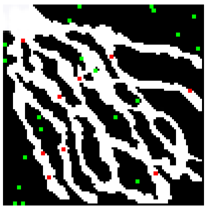

Fig 11c Stochastic realizations of the complex sedimentary model: (c) realization using optimized MPG facies modeling based on a DFNN
